# Faculty’s work engagement in patient care: impact on job crafting of the teacher tasks

**DOI:** 10.1186/s12909-018-1411-z

**Published:** 2018-12-19

**Authors:** Joost W. van den Berg, Christel P. M. Verberg, Albert J. J. A. Scherpbier, A. Debbie C. Jaarsma, Onyebuchi A. Arah, Kiki M. J. M. H. Lombarts

**Affiliations:** 1 Professional Performance research group, Department of Medical Psychology, Academic Medical Center (AMC-UvA), Meibergdreef 15, 1105AZ Amsterdam, the Netherlands; 2 ICLON, Leiden University Graduate School of Teaching, Leiden, the Netherlands; 3 Faculty of Health, Medicine and Life Sciences, Maastricht University, Maastricht, the Netherlands; 4 Center for Research & Innovation in Medical Education, University Medical Center Groningen, University of Groningen, Groningen, The Netherlands; 5 Department of Epidemiology, UCLA Fielding School of Public Health, Los Angeles, California USA

**Keywords:** Work engagement, Job crafting, Career development, Faculty development, Clinical teaching

## Abstract

**Background:**

High levels of work engagement protect against burnout. This can be supported through the work environment and by faculty themselves when they try to improve their work environment. As a result, they can become more engaged and better performers. We studied the relationship between adaptations by physicians to improve their teaching work environment, known as job crafting, and their energy levels, or work engagement, in their work as care provider and teacher. Job crafting encompasses seeking social (i) and structural (ii) resources and challenges (iii) and avoiding hindrances (iv).

**Methods:**

We established a cross-sectional questionnaire survey in a cohort of physicians participating in classroom and clinical teaching. Job crafting and work engagement were measured separately for physicians’ clinical and teaching activities. We analyzed our data using structural equation modelling controlling for age, gender, perceived levels of autonomy and participation in decision making.

**Results:**

383 physicians were included. Physicians’ work engagement for patient care was negatively associated with two job crafting behaviors in the teaching roles: seeking structural resources (classroom teaching: ß = − 0.220 [95% CI: -0.319 to − 0.129]; clinical teaching: ß = − 0.148 [95% CI: -0.255 to − 0.042]); seeking challenges (classroom teaching: ß = − 0.215 [95% CI: -0.317 to − 0.113]; clinical teaching:, ß = − 0.190 [95% CI: -0.319 to − 0.061]). Seeking social resources and avoiding hindrances were unaffected by physicians’ work engagement for patient care.

**Conclusions:**

High engagement for *teaching* leads to job crafting in teaching. High engagement for *patient care* does not lead to job crafting in teaching.

**Electronic supplementary material:**

The online version of this article (10.1186/s12909-018-1411-z) contains supplementary material, which is available to authorized users.

## Background

Faculty’s burnout is a major concern for the quality of clinical training, affecting students, residents and clinical faculty alike [1–4]. The negative effects of burnout include absenteeism from work, poor health and poor performance. Work engagement has been established as a positive opposite of burnout [5], within the framework provided by the Job Demands-Resources model [6]. Demands and resources refer to those work and personal characteristics which influence well-being at work. Under the right circumstances, resources boost positive well-being, or engagement, whereas demands drive negative well-being, or burnout [7]. It has been argued and evidenced that an increase in work engagement has a preventive effect on burnout [5], while increasing or maintaining performance in patient care [8] and teaching [9]. Table 1 highlights the commonalities and differences between burnout and work engagement [5, 10, 11]. Institutions can enable their clinical faculty to become engaged by providing a supportive work environment consisting of known job resources, such as ensuring the right amount of autonomy and enough participation in decision making [12].

**Table 1:** Commonalities and differences between burnout and work engagement

Characteristic	*Burnout*	*Work engagement*	*Meaning*
Level of activation	Exhaustion or low activation	Vigor or high activation	Burnout and work engagement represent ends of the activation spectrum
Level of identification	Cynicism or low identification	Dedication or high identification	Burnout and work engagement represent ends of the identification spectrum
Level of efficacy	Low efficacy	No opposite or equivalent	Burnout is associated with low levels of personal accomplishment
Level of absorption	No opposite or equivalent	High absorption	Work engagement is associated with high levels of concentration and engrossment
Summary of relationship:	Demands in work (e.g. time pressure) increase the risk of burnout. Resources in work (e.g. perceived autonomy) help overcome these demands and drive work engagement. When vigor and dedication are high, a buffer towards exhaustion and cynicism is maintained. High absorption additionally helps to perform well. (A. B. Bakker, 2011b; Maslach et al., 2001; Schaufeli, Salanova, González-Romá, & Bakker, 2002b)		

There is currently a gap in the literature on what clinical faculty do themselves to ensure they become or stay engaged. This knowledge will help us further understand how to guide them in these activities, in addition to what is known about how to optimize their work environment. From other contexts we know professionals shape their work environment on their own, to meet their needs and preferences in the perceived job demands and resources, called job crafting [13]. Job crafting leads professionals to be more engaged in their work and ultimately perform better [13–17]. Job crafting covers four strategies: (i) seeking social resources, (ii) seeking structural resources, (iii) seeking challenges or (iv) avoiding hindering demands. As such, job crafting involves changing both content of work as well as the work environment.

Crafting ones job is especially relevant for clinical faculty. Their concurrent responsibilities in patient care and teaching (in addition to any other responsibilities such as in research) require clinical faculty to find a balance in the various roles – under increasing expectations to perform highly in all roles [18]. A risk for burnout arises when faculty cannot spend enough time in the role most meaningful to them [2] or feel unsupported for either role [19]. However, for some faculty working in both roles may serve as a welcome change in pace, giving them energy and offering challenges, possibly leading to engagement to their work [20].

Clinical faculty experience different levels of work engagement in the roles of care provider and teacher; in general, physicians seem most engaged with patient care [9]. As a consequence, they work more energetically and more concentrated in patient care than in teaching. It may not necessarily mean faculty invest this energy in their teaching-role as well. Since faculty engaged to their work as care provider are not necessarily seen as better clinical teachers by residents [9], it remains uncertain if patient care work engagement leads to job crafting in teaching.

Theory does predict an *intra*-role effect between work engagement and job crafting: patient care work engagement will lead to job crafting within patient care practice [14]. What remains unknown is the *inter*-role effect: whether or not patient care work engagement additionally leads to job crafting in teaching, i.e. whether or not clinical faculty will invest any high levels of energy from patient care work into shaping their teaching work. This insight will help understand how clinical faculty find a balance between patient care and teaching and what institutions may contribute in this process. This may translate into policies and individual initiatives to create a better balance between roles and could provide coaches, faculty developers and similar mentor-like staff with better tools to aid faculty in their work.

**Fig. 1:**
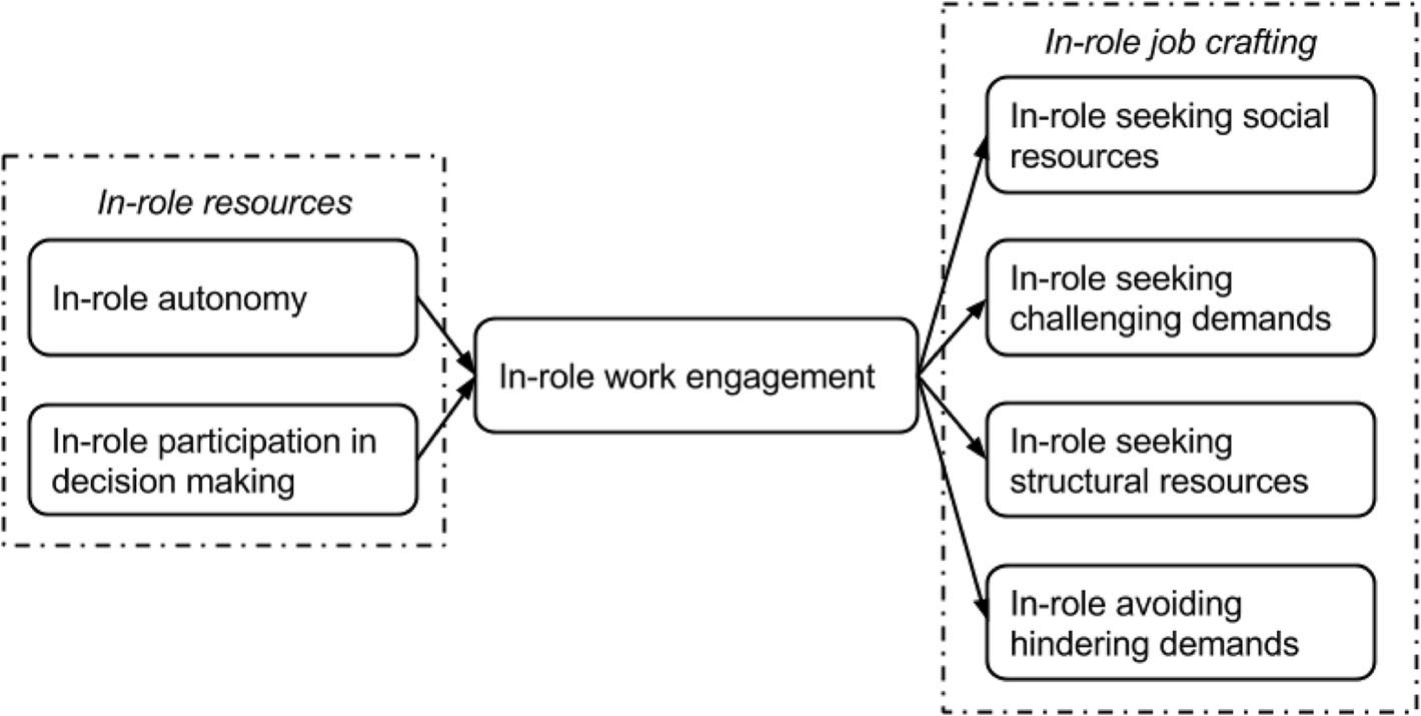
Visualization of our research questions indicating effects of work engagement on job crafting within and across roles. This figure details the model for each role separately

**Fig. 2:**
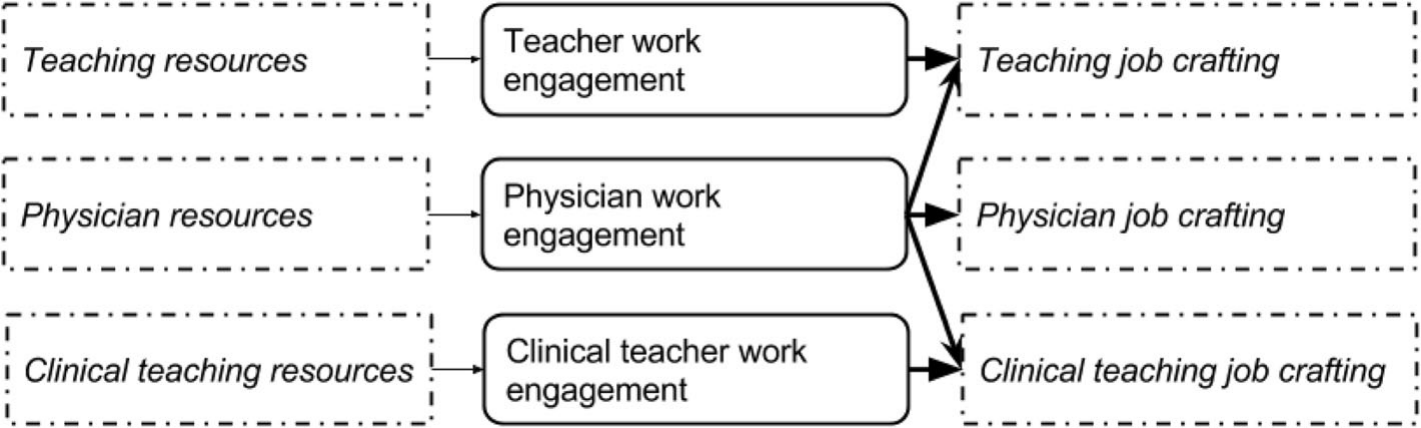
Visualization of our research questions indicating effects of work engagement on job crafting within and across roles. This model details the suggested relationship between roles with the resources and job crafting subscales displayed in a condensed way

To understand the relations between work engagement, job crafting and combining patient care with teaching, we chose to distinguish between classroom and clinical teaching to acknowledge the different settings in which both occur. Clinical teaching and patient care are often provided simultaneously whereas clinical faculty remove themselves from the clinical workplace to classrooms or lecture halls for ‘regular’ classroom teaching. We investigated two related research questions:

1. What is the impact of work engagement on job crafting *within* faculty’s roles in patient care, classroom teaching and clinical teaching? (Fig. 1).
2. What is the impact of patient care work engagement on job crafting in classroom teaching and clinical teaching practices, as an *across* roles effect? (Fig. 2) To answer both questions, we established a cross-sectional multi-center questionnaire survey in a cohort of clinical faculty combining patient care delivery with either classroom teaching or clinical teaching. For the separate activities of patient care, clinical teaching and classroom teaching, we assessed faculty’s work engagement and job crafting behaviors through validated instruments. We examined the relationship between work engagement and job crafting within and across patient care, classroom teaching and clinical teaching.

**Fig. 3:**
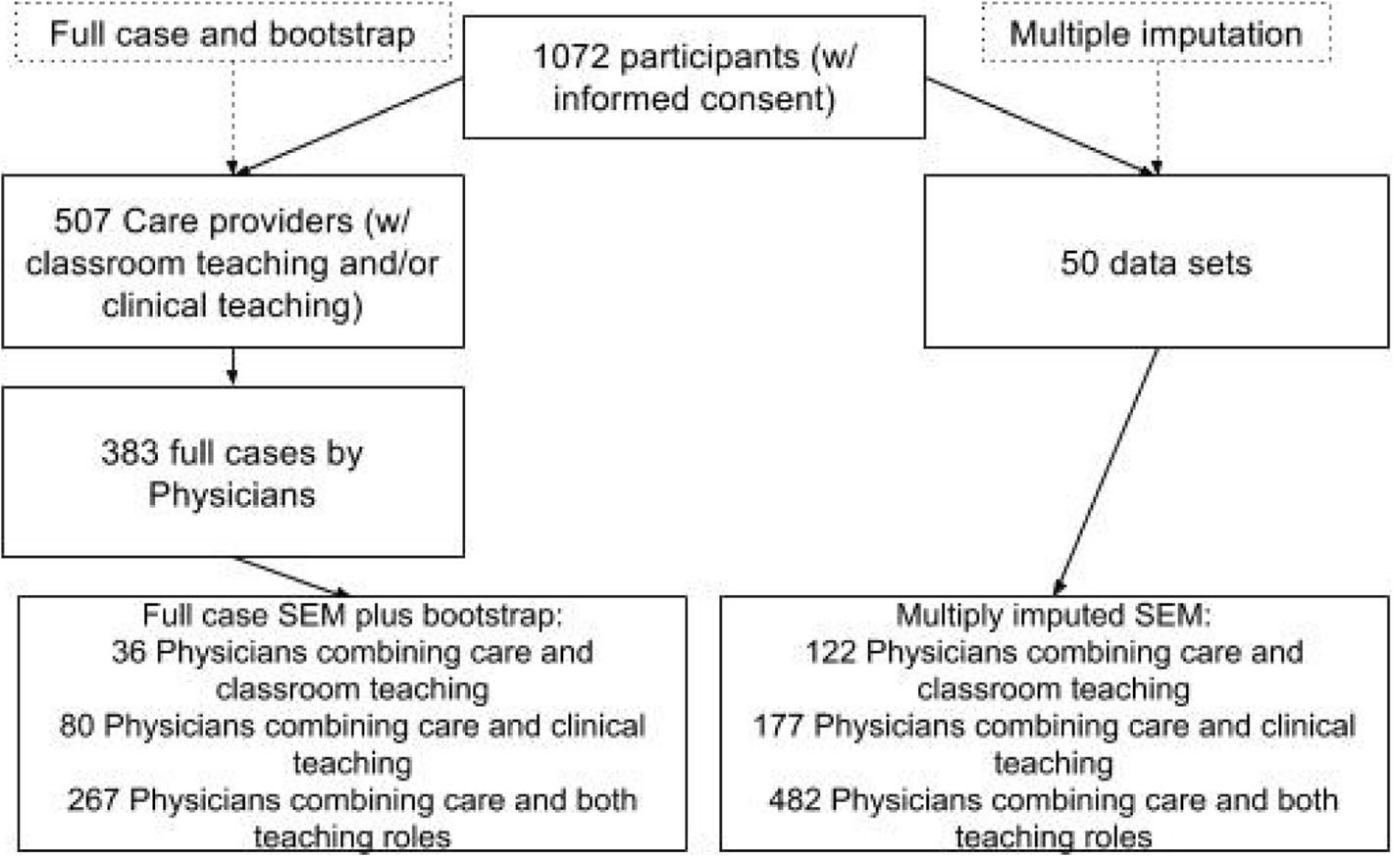
Sample flow chart and data handling

## Methods

### Design

We established a cohort of physicians participating in classroom teaching and clinical teaching and used an online questionnaire containing validated measures to quantify well-being, job crafting and additional variables.

### Setting and participants

In the Netherlands, all hospital physicians are expected to play an active role in teaching residents and interns (undergraduate students in the final three years of their six-year medical studies). Equally, residents are expected to actively participate in clinical teaching to interns. Both faculty and residents were included in our sample. Physicians at university teaching hospitals often also participate in undergraduate or bachelor level medical education, which typically involves classroom teaching. Lastly, especially in university teaching hospitals but generally in any teaching hospital as well, physicians participate in research to a certain extent.

Five academic medical centers and nine non-academic teaching hospitals from across the Netherlands participated in this study. Invitations for participation were sent by email, in which confidentiality was guaranteed and additional information was presented. Where possible a direct email was sent to physicians and university faculty, otherwise invitations were sent through snowballing to volunteers via administrators, course directors or secretarial offices. Data collection took place between February and August 2015.

### Ethics

Ethical approval was obtained through the Dutch Association for Medical Education Ethical Review Board under dossier number 385. Participant consent was written (in digital form) and without consent participation in the online survey was impossible.

### Questionnaires and predictive measures

A digital questionnaire was developed centered around three validated measures: the job crafting questionnaire [21], the work engagement questionnaire (UWES-9) [22] and a scale for the perceived job resources autonomy and participation in decision making [23]. These measures have been validated in different contexts, occupations, and cultures. In addition, they have previously been used in health care and education research specifically [9, 15, 24]. In total, these measures encompass 37 questions and participants were asked to fill out all questions for two or three of their professional roles depending on their work responsibilities at that time: classroom teacher, clinical teacher and patient care provider.

The main predictor was defined as the mean score on the UWES-9 questionnaire. The UWES-9 includes statements on the participant’s well-being and its nine items are rated on a zero to six scale representing ‘never’ to ‘always/daily’. An example statement is: “My work inspires me.” The statements cover feelings arising from and attitudes towards work and cover all three dimensions of work engagement (vigor, dedication and absorption).

The UWES-9 scores were in turn predicted by the mean score on the autonomy and participation in decision making scales. The autonomy-scale consists of three items. An example question is: “Can you decide on your own how to do your work?” The participation in decision making scale consists of four items. An example statement is: “I have a sufficient say in the working schedule.” The job resource-scales are rated on a regular five-point Likert scale, representing ‘never’ (1) to ‘often’ (5).

### Outcome measures: Job crafting

The main outcomes were defined as the mean scores on the four subscales of the job crafting questionnaire. Twenty-one statements cover the four subscales: (i) strengthening either social (five items) or (ii) structural resources (five items) in work, (iii) seeking challenges (five items) or (iv) the avoidance of hindering demands (six items). Example statements for each subscale are respectively: (i) “I ask others for feedback on my job performances.”, (ii) “I try to develop my capabilities.”, (iii) “If there are new developments, I am one of the first to learn about them and try them out.” and (iv) I make sure that my work is mentally less intense. The job crafting questionnaire is self-rated on a regular five-point scale and represents job crafting on a ‘never’ (1) to ‘often’ (5) basis. The statements cover tendencies and actual behaviors but do not address perceived success in job crafting.

### Covariates

Age and gender were added to the analysis to correct for possible confounding. The need to include hospital as covariate was tested based on the intra-class correlation.

To account for differences between university teaching hospitals (UTH) and general teaching hospitals (GTH) such as time allocated for teaching, this covariate was additionally included as binary variable in the analyses to correct for confounding.

To account for differences in needs and perceptions towards work engagement and job crafting, this covariate was additionally included as binary variable in the analyses to correct for confounding. Additionally, ANOVA was conducted on the work engagement and job crafting scores to determine whether both groups differed in mean values.

### Statistical analysis

We chose path analysis with structural equation modeling (SEM) to allow for simultaneous assessment of the interrelations between job resources, work engagement and job crafting within and across roles, given other variables.

We calculated Cronbach’s alpha on all scales and subscales separately as measure of reliability in our specific sample. The preparation for conducting the structural equation modeling then consisted of defining the structural model based on theory and our research question and resulted in the conceptual framework (Figs. 1 and 2) presented in the introduction. Subsequently, we checked the assumptions of normality, for outliers and for co-linearity and correlations between specified variables. For final variable selection, we calculated the intra-class correlation of the hospital variable to inform inclusion. The outcomes were corrected for age, gender, UTH versus GTH and medical specialist versus resident. We first performed our analysis with full-cases only and subsequently with multiple imputation using multivariate imputations by chained equations to deal with missing data. We used 50 imputations with 200 iterations based on the column with the highest percentage of missing data [25]. We used predictive mean matching for all columns. We bootstrapped the standard errors using 10,000 draws on complete cases only. We used the robust maximum-likelihood estimation method in the analysis as well as weighted least square estimation of mean and variance. The latter was used a sensitivity analysis to assess whether work engagement and job crafting would have a different relationship if these variables were to be considered ordered categorical variables. The results did not change when using weighted least square estimations of mean and variance. The relations between the predictors and outcomes in terms of their effect direction and size were reported using standardized coefficients with 95% confidence intervals.

Fit measures were calculated, but models were not re-specified based on these measures as our intent was to study specific path coefficients rather than analyzing full models.

R 3.2.3 [26] was used to prepare data and the *lavaan*-package [27] was used to handle the SEM analysis. The *semTools* [28] and *mice* [29] packages were used for imputation and analysis of missing data.

## Results

### Demographics

In total, 1072 participants representing 14 teaching hospitals started answering the questionnaire and gave informed consent, an additional 17 did not provide informed consent whose data were thus not included in our final database. Response per center ranged from 8 to 193; exact response rates were incalculable because of uncertainty about the exact extent of the teaching staff at most participating centers. Based on local estimations of the extent of the teaching staff and our responses, response was at most 20%.

Out of the 1072 participants, 507 reported that their most time consuming role, besides (classroom or clinical) teaching, was patient care (other options were research and management (which were mostly a secondary role) or teaching only). Out of these 507, 419 filled out the entire questionnaire and 383 of these were physicians, resulting in 383 measures for patient care work engagement and job crafting, 303 for classroom teaching and 347 for clinical teaching (Fig. 3). Excluded participants were full-time researchers, teachers or otherwise not involved with patient care. Full sample details and descriptive statistics are provided in Table 2.

**Table 2:** Sample characteristics and descriptive statistics (*N* = 383) A: other includes f.e. full professors, B: other includes f.e. specialized outpatient clinics, C: other includes f.e. primary care physicians

Gender	185 male (48.3%) and 198 (51.7%) female			
Age, in years	26–67 (median: 42.00)			
Employed as medical specialist / resident / other^A^	255 (66.6%) / 117 (30.5%) / 11 (2.9%)			
Employed at university teaching hospital (UTH) / general teaching hospital (GTH) / other^B^	177 (46.2%) / 183 (47.8%) / 23 (6.0%)	Range per hospital 7–60		
Primary teaching role in classroom teaching / clinical teaching / both	36 (9.4%) / 80 (20.9%) / 267 (69.7%)			
Working in surgical specialty / medical specialty / diagnostic or other^C^	99 (25.8%) / 145 (37.9%) / 139 (36.3%)			
Variable	Role	Sample mean (SD)	Medical specialist mean (SD) (*N* = 255)	Resident mean (SD) (*N* = 117)
Work engagement	Patient care	4.24 (0.90)	4.26 (0.94)	4.25(0.80)
	Clinical Teaching	3.93 (0.97)	4.05 (0.92)	3.64 (1.06) (*p* < 0.001)
	Classroom Teaching	3.78 (1.07)	3.80 (1.09)	3.71 (1.04)
Job Crafting – Seeking structural Resources	Patient care	4.02 (0.50)	3.97 (0.51)	4.11 (0.49) (*p* = 0.01)
	Clinical Teaching	3.41 (0.63)	2.21 (0.59)	2.48 (0.66) (*p* < 0.001)
	Classroom Teaching	3.29 (0.68)	3.24 (0.66)	3.33 (0.69)
Job Crafting – Seeking social Resources	Patient care	2.64 (0.77)	2.34 (0.60)	3.37 (0.66) (*p* < 0.001)
	Clinical Teaching	2.30 (0.62)	3.46 (0.61)	3.19 (0.63) (*p* < 0.001)
	Classroom Teaching	2.26 (0.66)	2.03 (0.55)	2.68 (0.70) (*p* < 0.001)
Job Crafting – Seeking challenges	Patient care	2.89 (0.65)	2.89 (0.63)	2.89 (0.71)
	Clinical Teaching	2.51 (0.72)	2.55 (0.70)	2.37 (0.78) (*p* < 0.05)
	Classroom Teaching	2.41 (0.70)	2.35 (0.65)	2.50 (0.77)
Job Crafting – Avoiding hindrances	Patient care	1.49 (0.46)	1.44 (0.40)	1.58 (0.53) (*p* < 0.01)
	Clinical Teaching	1.43 (0.44)	1.42 (0.40)	1.44 (0.45)
	Classroom Teaching	1.45 (0.47)	1.43 (0.47)	1.49 (0.46)
Autonomy	Patient care	3.28 (0.84)	3.52 (0.77)	2.70 (0.72) (*p* < 0.001)
	Clinical Teaching	3.37 (0.85)	3.54 (0.79)	2.86 (0.81) (*p* < 0.001)
	Classroom Teaching	3.14 (0.93)	3.28 (0.97)	2.84 (0.81) (*p* < 0.001)
Participation in Decision Making	Patient care	3.35 (0.90)	3.62 (0.83)	2.71 (0.72) (*p* < 0.001)
	Clinical Teaching	3.22 (1.00)	3.48 (0.90)	2.45 (0.86) (*p* < 0.001)
	Classroom Teaching	3.00 (0.97)	3.21 (0.99)	2.53 (0.80) (*p* < 0.001)

Individual hospitals were not included as a variable in our final model because the confidence interval of the intra-class correlation (ICC) for all measures (except for autonomy as classroom teacher) was covering zero (i.e. the lower bounds were negative and upper bounds positive), indicating no influence from location. Cronbach’s alpha’s of all measures used ranged from 0.64 (for job crafting structural resources in patient care) to 0.92 (for work engagement in classroom teaching). We consider these reliability levels moderate to very good.

### Intra-role impact of work engagement on job crafting

Clinical faculty experience different levels of work engagement in the roles of care provider and teacher. The mean level of work engagement for patient care was 4.24 (on a 0 to 6 scale), for clinical teaching 3.93 and classroom teaching 3.78. Tables 3 and 4 show correlations between engagement for patient care, classroom teaching (Table 3) and clinical teaching (Table 4) and job crafting (JC) in these roles. First, regarding the intra-role effects, there was a consistent positive effect of work engagement on JC subscale ‘seeking structural resources’, ‘social resources and challenges’ *within roles*. Within classroom teaching, the strongest effect was found when classroom teaching was combined only with patient care (and not also classroom teaching), for ‘seeking of structural resources’ (ß = 0.480, 95% CI: 0.126 to 0.834). There was a minor negative effect of classroom teacher work engagement on the JC subscale ‘avoidance of hindering demands in classroom teaching’ (ß = − 0.062, 95% CI: -0.105 to − 0.019, for clinical faculty combining both teaching roles with patient care). Within clinical teaching, the strongest effect was found when clinical teaching was combined only with patient care (and not also classroom teaching), again for the JC domain ‘seeking structural resources’ (ß = 0.379. 95% CI: 0.252 to 0.506). There was again a minor negative effect of clinical teacher work engagement on ‘avoidance of hindering demands in clinical teaching’ (ß -0.056, 95% CI: -0.099 to − 0.012, for physicians combining both teaching roles with patient care).

**Table 3:** Effects of work engagement for both patient care, classroom and clinical teaching on job crafting in classroom teaching (bold indicates findings with *p* < 0.05), full cases

Job crafting in classroom teaching	Work engagement with patient care		Work engagement with classroom teaching	
	Combined with…		Combined with…	
	… only classroom teaching (*N* = 36)	... both teaching roles (*N* = 267)	... only patient care (*N* = 36)	... clinical teaching and patient care (*N* = 267)
	Std. coef. (95% CI)	Std. coef. (95% CI)	Std. coef. (95% CI)	Std. coef. (95% CI)
Seeking social resources	ß −0.070 (− 0.369–0.228)	ß − 0.020 (− 0.115–0.076)	**ß 0.334 (0.019–0.650)**	**ß 0.137 (0.058–0.215)**
Seeking structural resources	ß − 0.123 (− 0.489–0.243)	**ß − 0.220 (− 0.319 – − 0.120)**	**ß 0.480 (0.126 - 0.834)**	**ß 0.361 (0.271–0.452)**
Seeking challenges	ß − 0.-0.179 (− 0.542–0.185)	**ß − 0.215 (− 0.317 – − 0.113)**	**ß 0.480 (0.079–0.882**	**ß 0.354 (0.274–0.434)**
Avoiding hindrances	ß −0.048 (− 0.287–0.191)	ß −0.006 (− 0.091–0.079)	ß 0.197 (− 0.060–0.454)	**ß − 0.062 (− 0.105 - -0.019)**

**Table 4:** Effects of work engagement for both patient care, classroom and clinical teaching on job crafting in clinical teaching (bold indicates findings with *p* < 0.05), full cases

Job crafting in clinical teaching	Work engagement with patient care		Work engagement with clinical teaching	
	Combined with…		Combined with…	
	… only clinical teaching (*N* = 80)	… both teaching roles (N = 267)	… only patient care (N = 80)	… classroom teaching and patient care (N = 267)
	Std. coef. (95% CI)	Std. coef. (95% CI)	Std. coef. (95% CI)	Std. coef. (95% CI)
Seeking social resources	ß −0.180 (− 0.366–0.006)	ß − 0.015 (− 0.135–0.104)	**ß 0.341 (0.187–0.495)**	**ß 0.183 (0.088–0.279)**
Seeking structural resources	ß − 0.072 (− 0.223 - 0.078)	**ß − 0.148 (− 0.255 - -0.042)**	**ß 0.379 (0.252–0.506)**	**ß 0.391 (0.290–0.491)**
Seeking challenges	ß − 0.194 (− 0.422–0.034)	**ß − 0.190 (− 0.319 - -0.061)**	**ß 0.361 (0.138–0.583)**	**ß 0.428 (0.320–0.536)**
Avoiding hindrances	ß − 0.113 (− 0.227–0.001)	ß − 0.015 (− 0.088–0.059)	ß 0.049 (− 0.014–0.111)	**ß − 0.056 (− 0.099 - -0.012)**

The full results for the impact of patient care work engagement on patient care job crafting can be found in Additional file 1: Table S2. The effect of patient care working engagement on patient care job crafting was mostly positive, i.e. higher levels of work engagement were associated with increased job crafting behaviors. The strongest effect was found when participants combined work in patient care with classroom teaching only, for seeking challenges (ß = 0.348, 95% CI: 0.130–0.567). The effect of patient care work engagement on avoidance of demands was negative (ß = − 0.127, 95% CI: -0.186 to − 0.069) for clinical faculty combining work in both teaching roles with patient care.

**Fig. 4:**
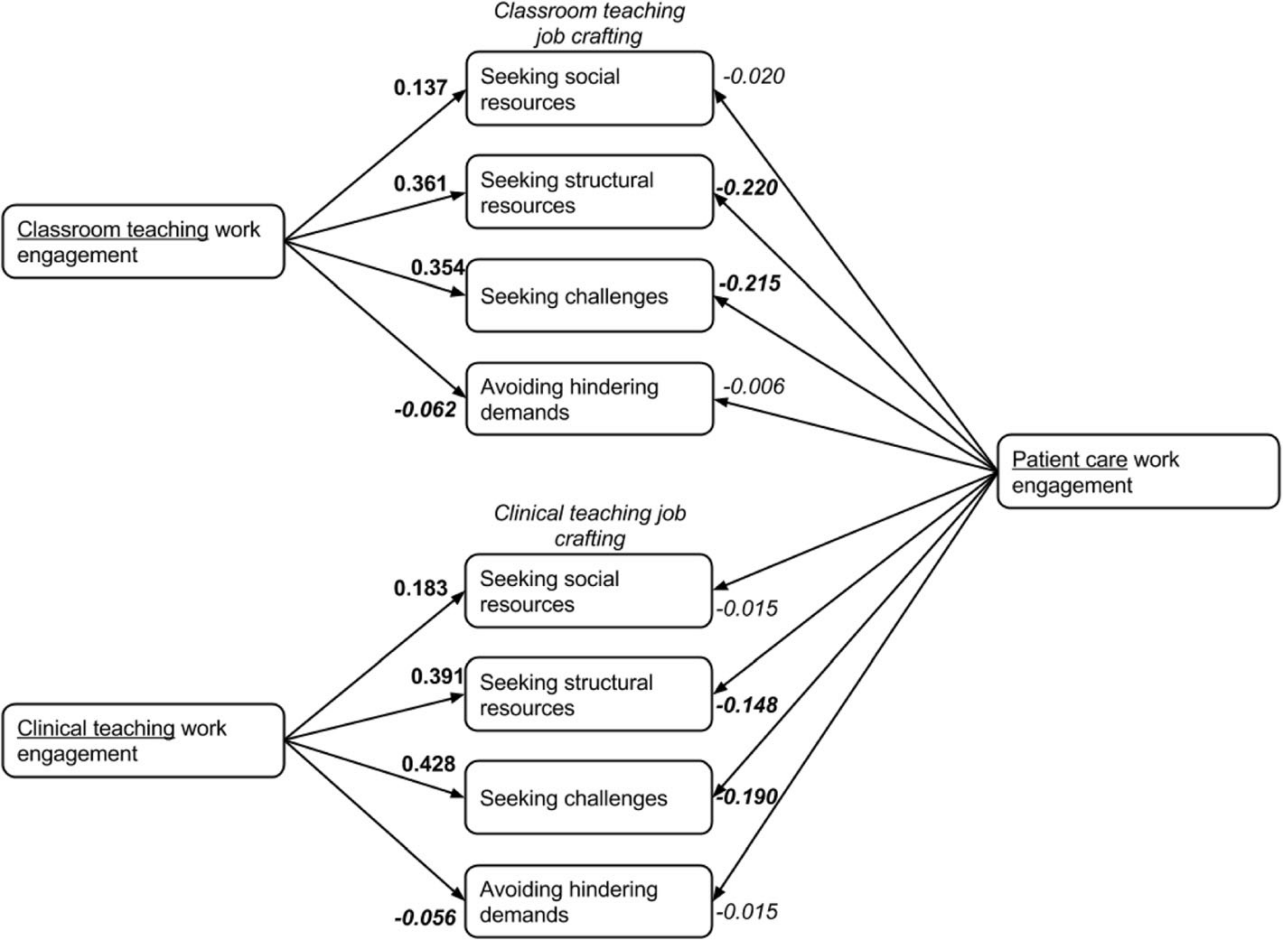
Results on primary aim of this study: effect of patient care work engagement on job crafting in clinical and classroom teaching. **Bold** indicates *p* < 0.05; *italics* indicate negative correlations (from Tables 3 and 4; physicians who combine patient care with both teaching roles)

Figure 4 summarizes the main findings from both the intra-role and inter-role effects.

### Inter-role impact of work engagement and job crafting

Second, on the inter-role effects, as we studied the impact of patient care work engagement on job crafting of the teaching tasks. The effect of patient care work engagement on classroom teaching and clinical teaching job crafting was consistently absent or negative. When clinical faculty combined patient care with both classroom and clinical teaching, there was a negative effect of being engaged for patient care on seeking structural resources and challenges in classroom teaching (ß = − 0.220, 95% CI: -0.319 to − 0.120, and ß = − 0.215, 95% CI: -0.317 to − 0.113 respectively) and clinical teaching (ß = − 0.148, 95% CI: -0.255 to − 0.042, and ß = − 0.190, 95% CI: -0.319 to − 0.061 respectively). Being engaged for patient care did not affect the JC subscales ‘seeking social resources’ and ‘avoiding hindrances’ in neither of the two teaching roles. These findings are reported in Tables 3 and 4.

The inter-role relationship from work engagement in teaching on job crafting in patient care showed a similar pattern to vice versa relationship described in the previous paragraph. Full details can be found in Table 5. Most correlations found were negative influences from teaching work engagement on patient care job crafting, mainly for classroom teaching work engagement on seeking challenges in patient care (ß = − 0.236, 95% CI: -0.398 to − 0.075). However, there appears to be a positive relationship with work engagement for clinical teaching on seeking challenges in patient care, both when clinical teaching was combined only with patient care (ß = 0.206, 95% CI: 0.031 to 0.381) and when combined with both teaching roles (ß = 0.131, 95% CI: 0.000 to 0.263).

**Table 5:** Effects of work engagement for both patient care, classroom and clinical teaching on job crafting in patient care (bold indicates findings with *p* < 0.05), full cases

Job crafting in patient care	Work engagement with classroom teaching		Work engagement with clinical teaching	
	Combined with…		Combined with…	
	… only patient care	… clinical teaching and patient care	… only patient care	… classroom teaching and patient care
	Std. coef. (95% CI)	Std. coef. (95% CI)	Std. coef. (95% CI)	Std. coef. (95% CI)
Seeking social resources	ß −0.183 (− 0.407–0.041)	ß −0.016 (− 0.089–0.057)	ß −0.122 (− 0.290–0.045)	ß 0.048 (− 0.083–0.180)
Seeking structural resources	ß −0.113 (− 0.248–0.023)	**ß − 0.081 (− 0.160 - -0.002)**	ß −0.040 (− 0.144–0.064)	ß 0.094 (− 0.029–0.216)
Seeking challenges	**ß − 0.236 (− 0.398 - -0.075**	ß −0.015 (− 0.103–0.073)	**ß 0.206 (0.031–0.381)**	**ß 0.131 (0.000–0.263)**
Avoiding hindrances	**ß − 0.503 (− 0.749 - -0.258)**	ß 0.019 (−0.020–0.058)	ß 0.025 (− 0.084–0.134)	ß −0.034 (− 0.101–0.033)

### Impact of being a resident versus being a medical specialist

While medical specialists were more engaged for clinical teaching than residents were (mean 4.05 versus 3.64, *p* < 0.001), there were no difference for patient care and for classroom teaching.

However, in the structural equation model there appeared a stronger attempt from residents in seeking social resources in patient care (ß = 0.657, 95% CI: 0.399 to 0.916, for residents combining patient care with both teaching roles). For full details, see Table 6.

**Table 6:** Effects of being a resident on job crafting subscales (versus being a medical specialist) on job crafting in classroom teaching, clinical teaching and patient care (bold indicates findings with *p* < 0.05), full cases

Job crafting	Residents combining patient care with classroom teaching	Residents combining patient care with clinical teaching	Residents combining patient care with both teaching roles
	Std. coef. (95% CI)	Std. coef. (95% CI)	Std. coef. (95% CI)
*Patient care*			
Seeking social resources	**ß 1.052 (0.110–1.994)**	**ß 0.837 (0.403–1.272)**	**ß 0.657 (0.399–0.916)**
Seeking structural resources	ß − 0.073 (− 0.722–0.577)	ß 0.209 (− 0.169–0.588)	ß 0.122 (−0.060–0.303)
Seeking challenging demands	ß 0.571 (− 0.794–1.935)	**ß − 0.572 (− 1.021 - -0.123)**	ß 0.087 (− 0.169–0.343)
Avoiding hindering demands	ß 1.125 (−0.114–2.364)	ß −0.054 (− 0.364–0.256)	ß 0.064 (− 0.125–0.253)
*Classroom teaching*			
Seeking social resources	ß 0.074 (− 0.791–0.938)	N/A	**ß 0.372 (0.124–0.619)**
Seeking structural resources	ß −0.052 (− 0.888–0.784)	N/A	ß 0.097 (− 0.149–0.343)
Seeking challenging demands	ß 0.848 (− 0.029–1.726)	N/A	**ß 0.270 (0.014–0.525)**
Avoiding hindering demands	ß 0.075 (− 0.415–0.565)	N/A	ß 0.009 (−0.214–0.231)
*Clinical teaching*			
Seeking social resources	N/A	ß 0.097 (−0.321–0.515)	ß 0.098 (− 0.140–0.335)
Seeking structural resources	N/A	ß −0.225 (− 0.649–0.199)	ß −0.077 (− 0.311–0.157)
Seeking challenging demands	N/A	ß − 0.368 (− 0.787–0.051)	ß 0.177 (− 0.098–0.452)
Avoiding hindering demands	N/A	ß 0.044 (−0.207 0.295)	ß − 0.037 (− 0.245–0.170)

### Impact of autonomy and participation and decision making

Overall, the impact of the two job resources ‘autonomy’ and ‘participation in decision making’ on work engagement was mostly positive. When work in patient care was combined with work in both teaching roles, ‘participation in decision making’ was significantly correlated to being engaged for patient care, classroom and clinical teaching: ß = 0.229 (95% CI: 0.080 to 0.378), ß = 0.367 (95% CI: 0.194 to 0.539), ß = 0.314 (95% CI: 0.148 to 0.480) respectively. Autonomy was significantly correlated to patient care work engagement: ß = 0.163 (95% CI: 0.006 to 0.321). Autonomy was also correlated with work engagement for classroom teaching, but only when classroom teaching was combined with work in patient care: ß = 0.379 (95% CI: 0.037 to 0.721). The results for the correlation between resources in clinical teaching showed a similar pattern but were not statistically significant. The full results for the impact of autonomy and participation in decision making on job crafting can be found in Table 7.

**Table 7:** The impact of autonomy and participation in decision making on work engagement within roles (bold indicates findings with *p* < 0.05), full cases

Work engagement per combination of roles	Autonomy	Participation in decision making (PiDM)	Covariance between autonomy and PiDM
	Std. coef. (95% CI)	Std. coef. (95% CI)	Std. coef. (95% CI)
Patient care, only with classroom teaching	ß 0.165 (− 0.272–0.602)	ß 0.190 (− 0.222–0.602)	ß 0.434 (0.279–0.590)
Patient care, only with clinical teaching	ß 0.347 (− 0.014–0.707)	ß 0.112 (−0.160–0.384)	ß 0.589 (0.375–0.797)
Patient care, with both teaching roles	ß 0.163 (0.006–0.321)	ß 0.229 (0.080–0.378)	ß 0.534 (0.435–0.632)
Classroom teaching, with only patient care	ß 0.379 (0.037–0.721)	ß 0.209 (− 0.173–0.590)	ß 0.404 (0.195–0.613)
Classroom teaching, with clinical teaching and patient care	ß 0.182 (−0.002–0.366)	ß 0.367 (0.194–0.539)	ß 0.706 (0.591–0.821)
Clinical teaching, only with patient care	ß 0.319 (− 0.041–0.679)	ß 0.158 (− 0.126–0.442)	ß 0.674 (0.448–0.899)
Clinical teaching with classroom teaching and patient care	ß 0.148 (−0.038–0.335)	ß 0.314 (0.148–0.480)	ß 0.602 (0.503–0.702)

Overall, fit measures of the entire model were poor. For the SEM-model for physicians with only classroom teaching CFI was 0.748 and RMSEA was 0.193; physicians with only clinical teaching CFI was 0.721 and RMSEA 0.206; physicians with both teaching roles CFI was 0.722 and RMSEA was 0.191.

## Discussion

For faculty working in patient care, classroom teaching and/or clinical teaching, this study sought to investigate the relations between their work engagement and job crafting behaviours, both within each of the three professional roles (intra-role) as well as between those roles (inter-role relations). We found positive intra-role relationships between work engagement and job crafting, thus high engagement for either patient care or teaching leads to job crafting in patient care or teaching. Yet, between roles, this relation was absent or even negative. Thus, faculty who were more engaged for patient care were worse at adjusting their work in teaching than those who were less engaged (inter-role).

We suggest the psychological processes underlying work engagement may explain these findings. First, considering the characteristics of work engagement (i.e. vigor, dedication and absorption), it could be argued that being highly engaged makes it more difficult to detach oneself from the patient care role to invest time and resources in classroom teaching and clinical teaching. A possible, positive side effect then may be that patient care work engagement protects against distractions from other responsibilities, ensuring patient safety and quality of care. Secondly, clinical faculty may simply not recognize the benefit of seeking resources across roles in favor of avoiding time investment altogether but the low scores on the ‘avoiding hindering demands’ make this reasoning less likely.

The positive correlation between clinical teaching work engagement and seeking challenges in patient care, despite the other findings, could imply work engagement leads to seeking challenges within and across roles.

The finding that residents appear more inclined to seek social resources may result from seeking feedback being an element of social resources; residents are expected to seek feedback on their performance during residency training and this finding may reflect their intent to do so.

### Practical implications

Our results show that a high level of work engagement in a certain role leads to job crafting in that same role. Institutions can enable their clinical faculty to become engaged by providing a supportive work environment for each separate role. On the hospital level this could be embedded in both faculty development or wellbeing programs. On team level this could be part of jointly designing the best possible work environment for each team member.

The negative effects of higher work engagement on job crafting in teaching adds to the growing body of literature suggesting support for clinical faculty requires organizational changes and a personalized approach [30] and preferably equally across roles [19]. Separate career-tracks for clinician-educators have been advocated for decades [31], but these career-tracks aim to provide long term-support to those physicians who already chose to be an educator. Rather, we need to acknowledge that in current thinking on physician competence, being a (clinical) teacher is an integral part of any physician’s daily work – and many will not be able to adapt their work to fully meet their needs in all roles.

We have shown that providing autonomy and the opportunity to participate in decision making may boost engagement equally for patient care and teaching. In addition, it may be beneficial to provide clinical faculty with opportunities for professional development and provide them with feedback on their teaching performance [5], as has been suggested before [32]. A different approach could be to ensure teachers have the opportunity to connect with like-minded educators, to enable them to connect and tap into social resources informally [33]. To add to this, interventions specifically aimed at job crafting may be considered [34].

Lastly, we found intra-class correlation for hospitals to be low on both work engagement and job crafting subscales. This suggests support needs to be initiated at a lower organizational level, such as the department or more likely according to individual needs [20].

### Strengths and limitations

The main strength of this study is its multicenter design. This has increased generalizability and allowed us to assess variance within and between hospitals. In addition, the transformation from the physician as solely a provider of care to a medical professional who is expected to show competence in multiple roles is a global trend and tension between academic roles appears universal.

The main limitation of this study is its cross-sectional design. While it is known that work engagement and job crafting affect each other cyclically throughout time, its relation within and across roles have so far remained unstudied in this context. We consider this cross-sectional study as a necessary step towards a longitudinal design. Furthermore, the convenience sampling method leads to some uncertainty towards representativeness of our sample. Our sample is consciously diverse and this leads to wide ranges on all included variables and a wide variety in participants’ backgrounds. As such our findings may turn out to be more nuanced in specific populations within the medical education context.

Lastly, while exact response rates were unknown, there is a risk for nonresponse or selection bias. Several findings suggest there is a low risk these biases would strongly affect our outcomes. Baseline characteristics did not differ significantly between centers with a lower or higher absolute response on work engagement and job crafting. In addition, our work engagement scores and their distribution across roles is consistent with previous research [9, 35]. Furthermore, considering the consistent findings across subgroups, it is conceivable that more extreme responses would more likely inflate coefficients without essentially changing the negative correlation between work engagement and job crafting between roles. However, it will be important in future research to decrease this uncertainty by replicating our findings, aiming for high response rate and where possible accounting for nonresponse bias for instance by using the nonresponse bias analysis method [36].

### Future research

The broader topic of understanding how to best support clinical faculty may benefit from qualitative approaches. Quantitative approaches may not capture the intricacies as work engagement and job crafting depend highly on interaction between individuals and interaction with the work environment. Qualitative approaches are especially suitable for gaining a deeper understanding of the underlying mechanisms, as noted in another journal recently [37]. Any longitudinal studies on either burnout or work engagement should consider job crafting as a variable in their statistical analysis.

## Conclusion

When physicians experience high levels of work engagement towards patient care, they seem less inclined to modify their teacher tasks. For job crafting in teaching (both classroom and clinical) to occur, engagement for teaching appears a prerequisite. Role-specific job crafting interventions may be necessary to provide adequate support for the role of teacher.

## Additional file

Additional file 1: **Table S1.** a – Effects of work engagement for both patient care, classroom and clinical teaching on job crafting in classroom teaching (bold indicates findings with *p* < 0.05), imputed cases. **Table S1.** b – Effects of work engagement for both patient care, classroom and clinical teaching on job crafting in clinical teaching, imputed cases. **Table S2.** Effects of work engagement for patient care on job crafting within patient care. **Table S3.** The impact of autonomy and participation in decision making on work engagement within roles, imputed cases. (DOC 76 kb)

## Abbreviations

CFI: Confirmatory Fit Index
CI: Confidence Interval
ICC: Intra-class correlation
JC: Job crafting
RMSEA: Root Mean Square Error of Approximation
SEM: Structural Equation Model
UWES-9: Utrecht Work Engagement Scale-9
WE: Work Engagement
