# Supplementary material for: Faculty’s work engagement in patient care: impact on job crafting of the teacher tasks
Source: BMC Med Educ. 2018 Dec 19;18:312. doi: 10.1186/s12909-018-1411-z (PMC6300023; doi:10.1186/s12909-018-1411-z)
Supplement: Supplementary file 1 — Table S1. a – Effects of work engagement for both patient care, classroom and clinical teaching on job crafting in classroom teaching (bold indicates findings with p < 0.05), imputed cases. Table S1. b – Effects of work engagement for both patient care, classroom and clinical teaching on job crafting in clinical teaching, imputed cases. Table S2. Effects of work engagement for patient care on job crafting within patient care. Table S3. The impact of autonomy and participation in decision making on work engagement within roles, imputed cases. (DOC 76 kb) [file 12909_2018_1411_MOESM1_ESM.doc]

Additional file 1

Table S1a – Effects of work engagement for both patient care, classroom and clinical teaching on job crafting in classroom teaching (bold indicates findings with p <0.05), imputed cases

|  | **Work engagement with patient care**  **Combined with…** | | | **Work engagement with classroom teaching**  **Combined with…** | | |
| --- | --- | --- | --- | --- | --- | --- |
| **Job crafting in classroom teaching** |  | |  | | |  |
|  | **… only classroom teaching**  (N=36) | **… both teaching roles**  (N=267) | **… only patient care**  (N=36) | | **… clinical teaching and patient care** (N=267) |  |
|  | Std. coef. (95% CI) | Std. coef. (95% CI) | Std. coef. (95% CI) | | Std. coef. (95% CI) |  |
| **Seeking social resources** | ß 0.030  (-0.180 – 0.240) | ß -0.007  (-0.108 – 0.094) | ß 0.093  (-0.101 – 0.287) | | ß 0.101  (0.019 – 0.183) |  |
| **Seeking structural resources** | ß -0.017  (-0.310 – 0.277) | ß -0.118  (-0.259 – 0.023) | ß 0.134  (-0.122 – 0.391) | | ß 0.250  (0.132 – 0.368) |  |
| **Seeking challenges** | ß -0.023  (-0.223 – 0.176) | ß -0.101  (-0.212 – 0.010) | ß 0.112  (-0.067 – 0.292) | | **ß 0.227**  **(0.142 – 0.313)** |  |
| **Avoiding hindrances** | ß -0.003  (-0.136 – 0.130) | ß -0.013  (-0.085 – 0.059) | ß 0.024  (-0.103 – 0.151) | | ß -0.034  (-0.083 – 0.014) |  |

Table S1b – Effects of work engagement for both patient care, classroom and clinical teaching on job crafting in clinical teaching, imputed cases

| **Job crafting in clinical teaching** | **Work engagement with patient care**  **Combined with…** | | **Work engagement with clinical teaching**  **Combined with…** | |
| --- | --- | --- | --- | --- |
|  | **… only clinical teaching**  (N=80) | **… both teaching roles**  (N=267) | **… only patient care**  (N=80) | **… classroom teaching and patient care** (N=267) |
|  | Std. coef. (95% CI) | Std. coef. (95% CI) | Std. coef. (95% CI) | Std. coef. (95% CI) |
| **Seeking social resources** | ß -0.084 (-0.306 – 0.138) | ß -0.055 (-0.173 – 0.064) | ß 0.203 (-0.047 – 0.452) | **ß 0.173 (0.066 – 0.280)** |
| **Seeking structural resources** | ß -0.089 (-0.323 – 0.144) | ß -0.109 (-0.238 – 0.019) | **ß 0.308 (0.075 – 0.541)** | **ß 0.317 (0.194 – 0.441)** |
| **Seeking challenges** | ß -0.143 (-0.384 – 0.098) | ß -0.104 (-0.245 – 0.037) | **ß 0.252 (0.023 – 0.481)** | **ß 0.261 (0.133 – 0.388)** |
| **Avoiding hindrances** | ß -0.049 (-0.174 – 0.077) | ß -0.032 (-0.118 -0.055) | ß 0.000 (-0.098 – 0.098) | ß -0.026 (-0.089 – 0.038) |

Table S2 - Effects of work engagement for patient care on job crafting within patient care

|  |  | Work engagement for patient care |  |  |
| --- | --- | --- | --- | --- |
| Job crafting in patient care | Method | When combined only with classroom teaching | When combined only with clinical teaching | When combined with both teaching roles |
|  |  | Std. coef. (95% CI) | Std. coef. (95% CI) | Std. coef. (95% CI) |
| Seeking social resources | Full^a^ | ß 0.476 (0.214 – 0.738) | ß 0.152 (-0.009 – 0.313) | ß 0.118 (0.019 – 0.217) |
|  | MI^b^ | ß 0.196 (-0.059 – 0.452) | ß 0.110 (-0.077 – 0.296) | ß 0.094 (-0.020 – 0.209) |
| Seeking structural resources | Full | ß 0.287 (0.123 – 0.451) | ß 0.216 (0.085 – 0.348) | ß 0.245 (0.175 – 0.314) |
|  | MI | ß 0.240 (0.013 – 0.466) | ß 0.194 (0.033 – 0.355) | ß 0.213 (0.122 – 0.303) |
| Seeking challenges | Full | ß 0.491 (0.214 – 0.768) | ß 0.223 (0.065 – 0.381) | ß 0.216 (0.121 – 0.312) |
|  | MI | ß 0.208 (-0.049 – 0.466) | ß 0.186 (-0.003 – 0.375) | ß 0.185 (0.074 – 0.296) |
| Avoiding hindrances | Full | ß 0.208 (-0.169 - -0.585) | ß -0.066 (-0.177 – 0.044) | ß -0.140 (-0.201 – 0.078) |
|  | MI | ß -0.011 (-0.178 – 0.156) | ß -0.084 (-0.205 – 0.037) | ß -0.112 (-0.182 – -0.042) |
| ^a^ Complete cases  ^b^ Multiply imputed data |  |  |  |  |

Table S3 The impact of autonomy and participation in decision making on work engagement within roles, imputed cases

| Work engagement per combination of roles | Autonomy | Participation in decision making (PiDM) | Covariance between autonomy and PiDM |
| --- | --- | --- | --- |
|  | Std. coef. (95% CI) | Std. coef. (95% CI) | Std. coef. (95% CI) |
| Patient care, only with classroom teaching | ß 0.179 (-0.166 – 0.525) | ß 0.125 (-0.231 – 0.480) | 0.476 (0.193 – 0.760) |
| Patient care, only with clinical teaching | ß 0.245 (-0.053 – 0.543) | ß 0.116 (-0.159 – 0.391) | ß 0.585 (0.366 – 0.804) |
| Patient care, with both teaching roles | ß 0.185 (-0.007 – 0.378) | ß 0.173 (-0.006 – 0.353) | ß 0.538 (0.415 – 0.662) |
| Classroom teaching, with only patient care | ß 0.121 (-0.136 – 0.378) | ß 0.171 (-0.119 – 0.462) | ß 0.498 (0.227 – 0.769) |
| Classroom teaching, with clinical teaching and patient care | ß 0.117 (-0.044 – 0.277) | ß 0.273 (0.118 – 0.428) | ß 0.691 (0.566 – 0.815) |
| Clinical teaching, only with patient care | ß 0.278 (-0.033 – 0.589) | ß 0.116 (-0.148 – 0.379 | ß 0.660 (0.402 – 0.918) |
| Clinical teaching with classroom teaching and patient care | ß 0.184 (-0.044 – 0.277) | ß 0.222 (0.056 – 0.388) | ß 0.631 (0.500 – 0.761) |
